# Classification and segmentation of hip fractures in x-rays: highlighting fracture regions for interpretable diagnosis

**DOI:** 10.1186/s13244-025-01958-y

**Published:** 2025-04-15

**Authors:** Germán González, Joaquín Galant, José María Salinas, Emilia Benítez, Maria Dolores Sánchez-Valverde, Jorge Calbo, Nicolás Cerrolaza

**Affiliations:** 1 Robotics, Vision and Intelligent Technologies, Department of Computational Sciences and Artificial Intelligence, University of Alicante, Alicante, Spain; 2 Radiology Service, Hospital of San Juan de Alicante, Alicante, Spain; 3 IT Service, Hospital of San Juan de Alicante, Alicante, Spain; 4 Radiology Service, Hospital de la Vega Baja, Alicante, Spain; 5 Orthopedics Surgery, Hospital of San Juan de Alicante, Alicante, Spain

**Keywords:** Hip fractures, Artificial intelligence, X-rays, Image processing (Computer-assisted)

## Abstract

**Objective:**

To develop an artificial intelligence (AI) system capable of classifying and segmenting femoral fractures. To compare its performance against existing state-of-the-art methods.

**Methods:**

This Institutional Review Board (IRB)-approved retrospective study did not require informed consent. 10,308 hip x-rays from 2618 patients were retrieved from the hospital PACS. 986 were randomly selected for annotation and randomly split into training, validation, and test sets at the patient level. Two radiologists segmented and classified femoral fractures based on their location (femoral neck, pertrochanteric region, or subtrochanteric region) and grade, using the Evans and Garden scales for neck and pertrochanteric regions, respectively. A YOLOv8 segmentation convolutional neural network (CNN) was trained to generate fracture masks and indicate their class and grade. Classification CNNs were trained in the same dataset for method comparison.

**Results:**

On the test set, YOLOv8 achieved a Dice coefficient of 0.77 (95% CI: 0.56–0.98) for segmenting fractures, an accuracy of 86.2% (95% CI: 80.77–90.55) for classification and grading, and an AUC of 0.981 (95% CI: 0.965–0.997) for fracture detection. These metrics are on par with or exceed those of previously published AI methods, demonstrating the efficacy of our approach.

**Conclusions:**

The high accuracy and AUC values demonstrate the potential of the proposed neural network as a reliable tool in clinical settings. Further, it is the first to provide a precise segmentation of femoral fractures, as indicated by the Dice scores, which may enhance interpretability. A formal evaluation is planned to further assess its clinical applicability.

**Critical relevance statement:**

The proposed system offers high granularity in fracture classification and is the first to segment femoral fractures, ensuring interpretability.

**Key Points:**

- We present the first AI method that segments and grades femoral fractures.
- The method classifies fractures with fracture location and type.
- High accuracy and interpretability promise utility in clinical practice.

**Graphical Abstract:**

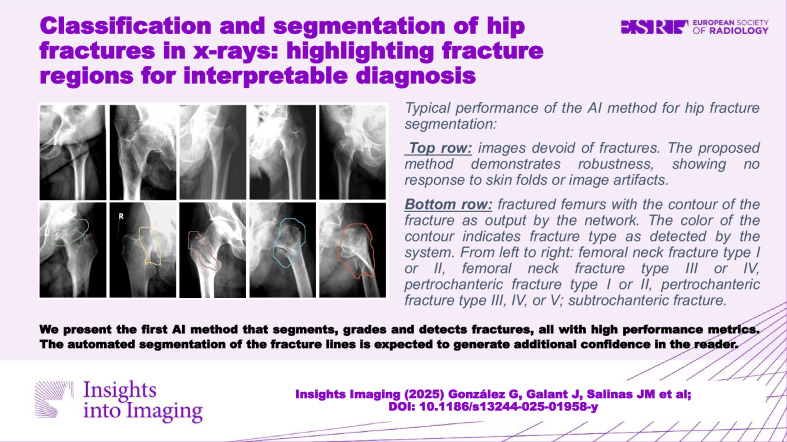

## Introduction

Hip fractures in elderly patients are a main cause of concern for health authorities. Although in recent years the mortality rate at 1-year post hip fracture has decreased from about 30% to 22% [1], they remain a leading cause of mortality and an important source of morbidity, with a loss of functional independence of up to 50% [2, 3].

Most fractures require surgical intervention, and earlier treatment significantly improves prognosis [4]. A non-insignificant number (4–12%) of patients have clinically relevant fractures that are not radiographically recognized on initial radiographs obtained in the emergency department [5–9]. Sensitivity and specificity vary significantly between general practitioners and first-year residents compared to attending radiologists and orthopedists, with average sensitivities of 69.2% and 73.1% for the first group and 96.2% for the second one [10]. Many unrecognized fractures on x-rays are caused by errors of perception [11], although a few fractures are impossible to detect with radiography.

First artificial intelligence methods for femoral fracture detection used classification convolutional neural networks to determine whether x-ray images depict fractures or not [10, 12–19]. All these methods achieved AUCs ranging between 0.91 and 0.99, but did not provide any information on the type or location of the fracture.

A second body of work extends binary classification to a multiclass classification method, where the output of the network is a label indicating the location of the fracture (i.e., neck, pertrochanteric, subtrochanteric) and sometimes whether the fracture is displaced or undisplaced [20–22]. For instance, Jiménez Sánchez et al [23], classify an image as normal or having a fracture in the head, neck or pertrochanteric area with 89% average accuracy; Yu et al [24] classify x-rays as being normal, having a subcapular fracture, a pertrochanteric fracture or a subtrochanteric fracture; Murphy et al [25] classify images as normal, trochanteric fracture or intracapsular fracture, with 92% accuracy; or Kitamura et al [26], who extends the classes to femoral and pelvic fractures, training a model for each of them, running them concomitantly and analyzing the response of the most likely model.

However, these methods uniquely provide a label for the whole image, and there is no indication of the regions or the evidence that the network used to provide the diagnosis. To address interpretability from classification networks, researchers resorted to heatmap generation techniques, such as Grad-Cam [27], to identify the pixels that most likely contributed to the network’s decision [20–22]. Recent publications have expanded on the concept of fracture localization by employing custom developed point networks [28] or object detection networks to draw bounding boxes around the pertrochanteric fractures [29].

Fracture detection and localization needs to be complemented with fracture grading, as the grade of the fracture determines its associated treatment [30]. Femoral neck fractures are graded with the Garden classification [31], pertrochanteric with the Evans-Jensen classification [32, 33] and subtrochanteric fractures with the Seinsheimer classification [34].

Only two studies have aimed to classify automatically fractures based on well-established grading scales [22, 35]. The work of Mutasa et al [35] focuses on classifying femoral neck fractures as normal, Garden I/II or Garden III/IV, achieving an accuracy of 86%. The work of Krogue et al [22] classifies femoral neck fractures as displaced or non-displaced mimicking grading in the scale of Garden.

The presented work differs from prior art in that we simultaneously detect, grade and segment hip fractures with a single neural network. With respect to grading, we extend the work of Mutasa et al [35] by including pertrochanteric and subtrochanteric fractures. We also extend the work of Krogue et al [22] by (1) adding subtrochanteric fractures to the study and (2) splitting pertrochanteric fractures into two classes: (1) type I and II and (2) type III, IV and V in Evans scale. Further, we are the only work that simultaneously grades and segments the fracture, providing clear information to the radiologist of the pixels that the network used to make the diagnosis.

## Methods

### Study design and data collection

Observational retrospective study approved by the Institutional Review Board (IRB) at Hospital Universitario de San Juan de Alicante (approval code: 21/060). Requirement for informed consent was waived.

The hospital information system (HIS) was queried for hospitalized patients with International Classification of Diseases (ICD) codes corresponding to all diagnoses of femoral fractures from 2010 to 2021. Upon retrieval of patient identifiers, the corporate picture archiving and communication system (PACS) was accessed to obtain the associated image studies. Images were anonymized using the management and labeling platform of the Medical Imaging Databank of the Valencia Region (BIMCV) [36].

A random subset of the studies was selected for annotation. This subset was further divided into training, validation and test sets. This division was performed randomly but ensuring that no images from the same subject appeared in more than one subset.

### Reference standard and image preprocessing

Selected images were labeled by two radiologists with 3 and 20 years of experience in interpreting emergency radiographs. The radiologists segmented each fracture by drawing a polygon around it, labeled it as either a femoral neck fracture, a pertrochanteric fracture, or a subtrochanteric fracture and graded it according to the Garden or Evans’ scales. Subtrochanteric fractures were not subjected to grading. Test images were reviewed by a committee comprising the initial radiologists and an additional specialized musculoskeletal radiologist with 20 years of experience. Labeling was conducted using the Open Health Imaging Foundation (OHIF) viewer [37], within the management platform of BIMCV.

Labels were aggregated to prevent excessive data fragmentation. Each femur was ultimately labeled as follows: (1) no fracture, (2) femoral neck fracture of Garden scale I or II, (3) femoral neck fracture of Garden scale III or IV, (4) pertrochanteric fracture of Evans scale I or II, (5) Pertrochanteric fracture of Evans scale III, IV, or V, or (6) subtrochanteric fracture.

Femurs were detected using a semi-automated method by training a YOLOv8 detection network [38] on bounding boxes centered in the femurs. Automated femoral detections underwent manual quality control, femur detections too close to the edge of the images or double detections were discarded. A 100% margin was added to each detection to incorporate surrounding information in the images, thereby forming femur ROIs. Femoral ROIs were cropped and resized to a canonical size while maintaining pixel isotropy. Cropped regions of interest, along with their associated fracture ROIs, formed the database used for fracture classification and segmentation.

### Femoral fracture segmentation and classification

A YOLOv8 image segmentation network [38] was trained on the ROIs. This network comprises three primary components: a backbone, a neck, and a decoupled head, each consisting of convolutional layers. The backbone is responsible for extracting features at various image resolutions, providing a hierarchical representation of the input data. The neck aggregates and refines these features to enhance object detection and segmentation capabilities. The decoupled head processes the features produced by the neck and, through distinct convolutional blocks, produces linked outputs for classification, detection, and segmentation tasks. The network architecture can be readily configured to perform any combination of these tasks simultaneously. The outputs are interlinked, ensuring that each segmentation is explicitly associated with a corresponding classification label.

As the YOLOv8 implementation only accepts 8-bit images, we scaled each image by adjusting its contrast between the 2% and the 98% percentile of pixel values and quantifying the image to the 0–255 range. The network was trained for 100 epochs, and the model with the best validation loss was saved.

We explored the relevance of model complexity and image size parameters, with the model complexity categorized as “nano,” “small,” or “medium,” and the image sizes set to 320^2^, 640^2^, 1024^2^, or 1536^2^ pixels. Each combination of parameters was trained six times, starting from different random seeds. We selected the combination of parameters that performed better on the validation set and, within those parameters, the model that had better validation accuracy to perform further evaluation.

### Evaluating baseline methods on the dataset

To replicate prior work and establish a reference performance metric on the dataset described in sections “Study design and data collection” and “Reference standard and image preprocessing,” we trained image classifiers that would associate the fracture label with each image.

Inspired by prior studies, we utilized five different network architectures: VGG16 [39], DenseNet121 [40], InceptionV3 [41], ResNet50V2 [42], and EfficientNetB4 [43]. Each architecture was used as a feature extractor, with the final layers of the network consisting of a global average pooling layer, a dropout layer with 20% probability of dropping weights and a final dense layer with six output classes (one per aggregated fracture type plus one for no fracture). Transfer learning was used, initializing the optimization of the convolutional layers with weights pre-trained on the ImageNet dataset [44]. The weights of the linear layers were initialized randomly.

Each network was trained six times. Standard data augmentation techniques were used: random horizontal flips, rotations, translations, zooming and brightness changes. Training was performed for 100 epochs, using the adaptive momentum optimizer [45] and categorical cross entropy as the loss function. Learning rate was halved if the validation accuracy did not improve for five consecutive epochs. The model with the lowest validation loss was saved. Image classification software was implemented in Python, using the TensorFlow library [46] for deep learning.

Prior work used small image sizes due to the complexity of the convolutional networks. To compare the performance of the segmentation model to prior work, we select the model with the best validation performance on the configuration “nano,” with the smallest image size (320^2^).

### Evaluation

The segmentation and baseline models were first evaluated on the test set for their capacity to discriminate between healthy and fractured femurs. The receiver operator characteristic (ROC) curve is measured and the area under it (AUC) reported. The model’s capacity to classify fractures, as described in the “Reference standard and image preprocessing” section, was evaluated using accuracy and confusion matrices. For the proposed segmentation models, we report the average Dice Similarity Coefficient (DSC) [47] between annotated fracture regions and the regions automatically detected. DSC measures the similarity between two binary masks by calculating twice the intersection area of the masks and dividing it by the sum of their individual areas.

### Data and statistical analysis

Evaluation and data analysis is performed using the scikit-learn library for data analysis [48] the pandas library for data management [49]. Confidence intervals of AUCs and comparison between paired ROC curves are computed with Delong’s methods as implemented in the pROC R library [50]. Confidence intervals for accuracies were obtained with the binomial proportion confidence interval method using the caret R library [51]. Comparisons between multiclass classifiers were made pairwise using the Durbin test, as implemented in the PMCMR R package [52]. *p*-values lower than 0.05 were considered statistically significant.

## Results

### Dataset

**Fig. 1:**
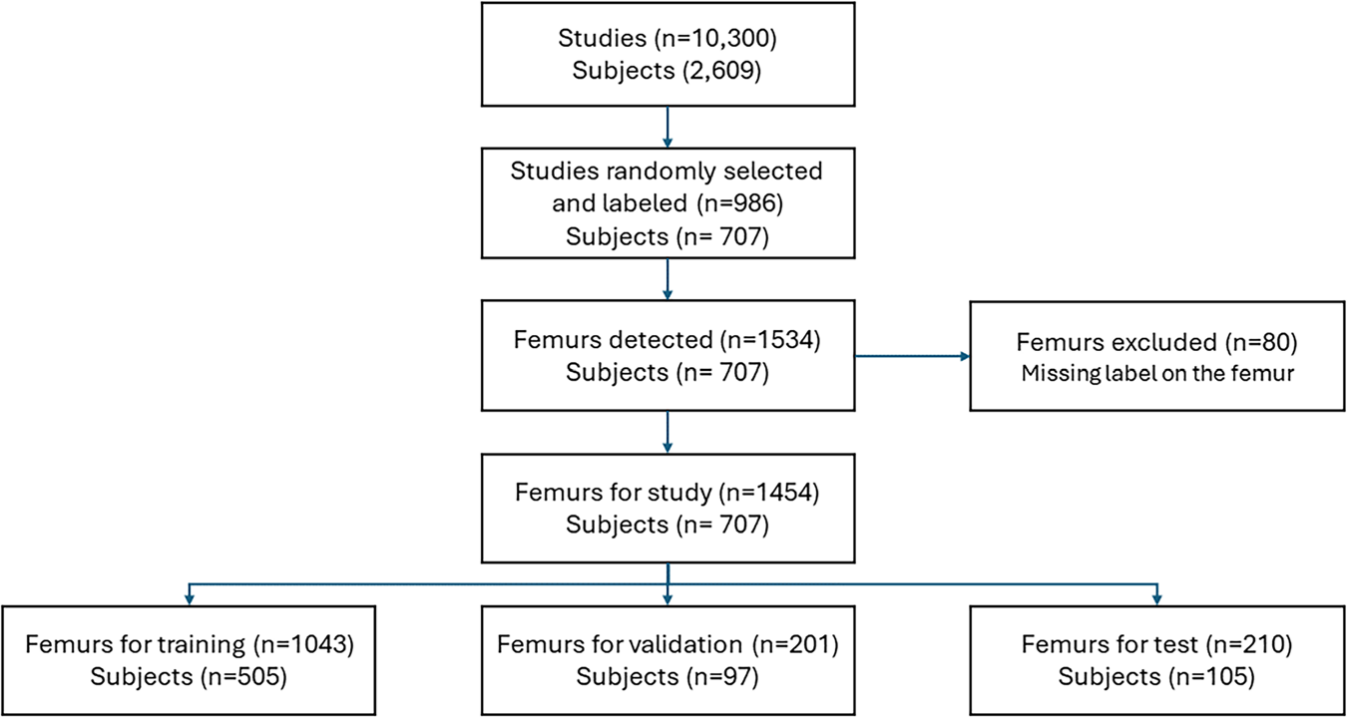
Structure of the study. 2609 subjects were included. Images of 707 of them were labeled. A semi-automated femoral detection method was used to detect femurs in the x-rays. Eighty femurs were excluded due to lack of manual labeling. The remaining femurs were split into train, validation and test sets at a patient level. No patient had images in two subsets

A summary of the dataset is shown in Fig. 1. A total of 10,300 images from 2609 patients were retrieved from the PACS. These images were acquired using five different devices from three manufacturers: Philips Medical Systems, Canon, and Agfa. The device models were Philips, DigitalDiagnost C99, Bucky Diagnost FS, Essenta DR, Agfa DR 100 and Canon M1 mobile x-ray unit.

986 hip x-ray studies from 707 patients were randomly selected for annotation. Most selected images were digital radiographies (DX, 70.9%) and a smaller proportion computed radiography (CR, 29.1%). The statistics of the annotated studies are presented in Table 1.

**Table 1:** Dataset quantitative details

	Total	Train	Validation	Test
Subjects	707	505	97	105
Age (mean, std)	81.8 (11.8)	81.9 (11.8)	82.3 (11.7)	80.9 (12.3)
Sex (female, %)	68.02	67.69	68.66	69.05
Studies	846	609	115	122
Study type (DX%)	70.9	70.37	72.14	72.38
Number of femurs	1454	1043	201	210
Fracture type				
No fracture	495	363	67	65
Neck. Garden I or II	84	57	11	16
Neck. Garden III or IV	337	254	42	41
Pertro. Evans I or II	205	134	37	34
Pertro. Evans III, IV or V	275	199	34	42
Subtrochanteric	58	36	10	12

1586 femurs were detected in those 986 hip x-rays. Three femurs were missed by the network and added manually. Femur detections were rejected for being very close to the edge and not containing the whole femur (*n* = 49), being a duplicated detection (*n* = 1) or not detecting a femur (*n* = 5). Eighty femurs were excluded as they were not annotated, as in some images, only one of the two visible femurs was labeled by the radiologist. The remaining 1454 femurs with their corresponding reference standard were split randomly into training (*n* = 1043 femurs from 505 patients), validation (*n* = 201 femurs from 97 patients) and test sets (*n* = 210 femurs from 105 patients). No patient had images on two different subsets. Dataset characteristics are shown in Table 1.

### Optimization of the segmentation model complexity and image size

Quantitative results analysis of the performance of the YOLOv8 method with respect to image size and model complexity are shown in Table 2. In general terms, AUC increased with image size. Accuracy increased with image size except for the very last image size, where it decreased in comparison to the previous one, and did not improve with model complexity. With respect to the Dice coefficient, lower image sizes obtain higher scores, with the maximum average score (0.780) obtained with the combination of a small model with image size of 640 × 640.

**Table 2:** Performance of the image segmentation networks on the test dataset

	Model/image size	320	640	1024	1536
AUC	Nano	0.978 (0.005)	0.983 (0.006)	0.985 (0.009)	0.980 (0.006)
	Small	0.979 (0.009)	0.985 (0.006)	0.986 (0.008)	**0.989** (**0.003)**
	Medium	0.980 (0.005)	0.985 (0.005)	0.985 (0.003)	0.983 (0.005)
Accuracy	Nano	81.11% (2.17)	82.54% (1.15)	83.17% (1.23)	80.55% (4.16)
	Small	79.68% (1.69)	81.98% (2.97)	**84.40%** (**2.51)**	81.90% (1.20)
	Medium	80.68% (1.57)	81.76% (2.32)	83.19% (1.89)	83.13% (1.57)
DICE	Nano	0.771 (0.023)	0.778 (0.016)	0.777 (0.014)	0.767 (0.018)
	Small	0.777 (0.009)	**0.780** (**0.011)**	0.761 (0.008)	0.755 (0.010)
	Medium	0.778 (0.014)	0.774 (0.015)	0.761 (0.012)	0.765 (0.006)

We explored two key parameters: image size and model complexity

Evaluation is performed in a binary classification aspect by measuring the AUC, in a multiclass classification setting by measuring the accuracy and in a segmentation setting by measuring the Dice coefficient. In this table, we report mean and standard deviation values for the six training runs. Bold numbers highlight best configuration. Underlined numbers highlight second best configuration

### Evaluation of a single segmentation model

We select the model *small* and image size of 1024 × 1024 as they show the highest average AUC and accuracy in the validation set (Table S1). From that configuration, we select the iteration with the highest validation accuracy. When evaluating its capacity to detect fractures on the test set, the model exhibits an AUC of 0.981 (95% CI [0.965–0.997]), classifying femurs as fractured or not with an accuracy of 96.2% (95% CI [92.6–98.3%]). Sensitivity, specificity, positive predictive value, and negative predictive value are 95.17%, 98.46%, 99.28%, and 90.14%, respectively.

**Fig. 2:**
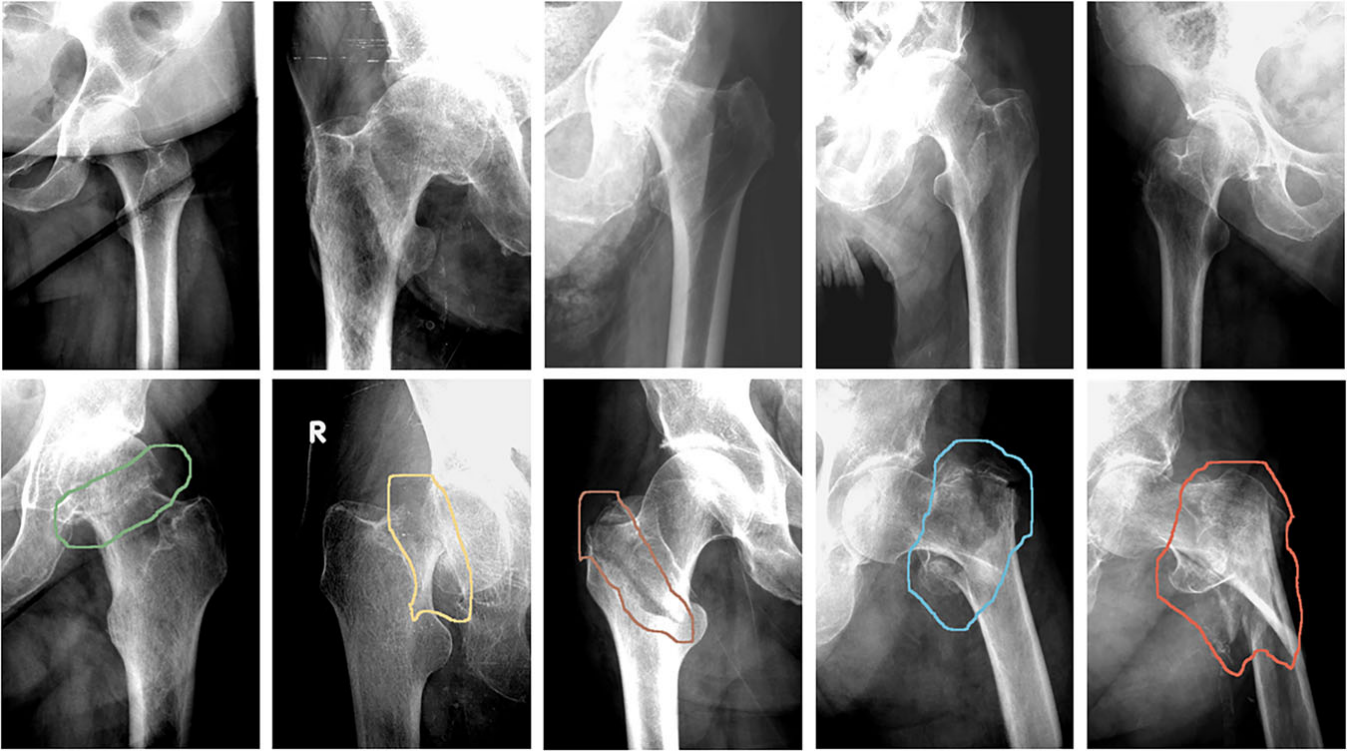
Typical performance of the network. Top row: images devoid of fractures. The proposed method demonstrates robustness, showing no response to skin folds or image artifacts. Bottom row: fractured femurs with the contour of the fracture as output by the network. The color of the contour indicates fracture type. From left to right: femoral neck fracture type I or II, femoral neck fracture type III or IV, pertrochanteric fracture type I or II, pertrochanteric fracture type III, IV, or V; subtrochanteric fracture

**Fig. 3:**
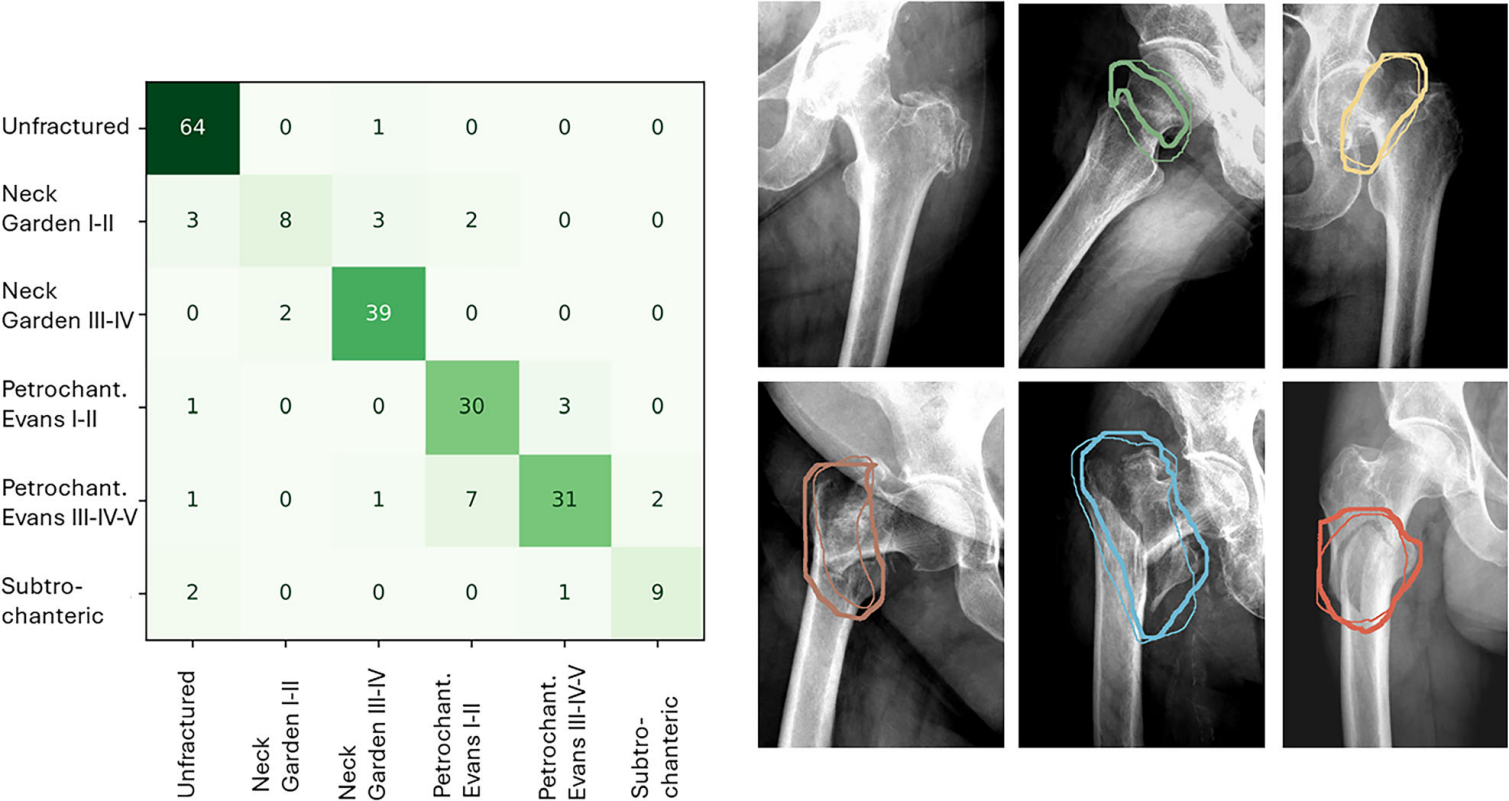
Performance of the model evaluated in the “Evaluation of a single segmentation model” section. Left: Confusion matrix for the model. Right: typical detections. Reference standard is shown with a thin line, segmentations with a thick line. Color encodes fracture type. Top row, from left to right: no fracture, femoral neck fracture of Garden type I or II, femoral neck fracture of Garden type III or IV. Bottom row from left to right: pertrochanteric fracture of Evans type I or II, pertrochanteric fracture of Evans type III, IV or IV, subtrochanteric fracture

When evaluating the model’s capacity to classify fractures according to the classification outlined in the “Reference standard and image preprocessing” section, it achieved an accuracy of 86.19% (95% CI [80.77–90.55]). Figure 2 shows typical responses of the network to femoral images. The confusion matrix for this model is shown in Fig. 3. Most classification errors (*n* = 15) occurred within the same fracture location, while six classification errors involved misclassifying the fracture location.

When evaluating the performance of the model to segment fractures, the model achieved an average Dice coefficient of 0.77 (95% CI [0.56–0.98]). Example images of the segmentation marked by the radiologist and the segmentations obtained by the model are shown in Fig. 3.

### Comparison to baseline models

We compared the performance of the YOLOv8 models to other neural networks used for femoral fracture detection. The results are shown in Table 3. All classification methods obtained high AUC scores when classifying a femoral image as fractured or healthy, ranging from an average AUC value of 0.993 [0.985–1.000] (DenseNet 121) to values of 0.981 [0.965–0.997] (YOLOv8). No statistically significant difference was found between models’ AUC.

**Table 3:** Performance of the image classification networks on the test dataset

	Performance on the test set		
Network	AUC [95% CI]	Accuracy [95% CI]	DICE [95% CI]
VGG16	0.986 [0.975–0.998]	74.29 [67.82–80.05]*	N/A
DenseNet121	0.993 [0.985–1.000]	83.81 [78.12–88.52]	N/A
EfficientNetB4	0.990 [0.981–0.998]	79.05 [72.91–84.34]	N/A
InceptionV3	0.985 [0.972–0.998]	77.62 [71.37–83.07]	N/A
ResNet50V2	0.982 [0.967–0.997]	80.00 [73.94–85.19]	N/A
Segm (“nano,” 320 × 320)	0.982 [0.970–0.995]	83.33 [77.59–88.11]	0.78 [0.57–0.98]
Segm (“small,” 1024 × 1024)	0.981 [0.965–0.997]	86.19 [80.77–90.55]*	0.77 [0.56–0.98]

From the six training runs, we selected the model with the highest validation accuracy

All models were statistically evaluated in pairs. No statistical difference was found for AUCs. The only statistically significant difference in accuracy was found between the VGG16 model and YOLOv8 (“small,” 1024^2^), both marked with an asterisk (*)

The accuracy of the baseline networks to classify fracture types ranged from 74.29% [67.82–80.05%] for VGG16 to 86.19% [80.77–90.55%] for YOLOv8. It is notable that networks with similar area under the curve (AUC) scores showed varying performance when classifying the specific type of fracture. The only significant difference in accuracies was found between VGG16 and YOLOv8 (small, 1024^2^).

## Discussion

We have trained a single artificial intelligence model that simultaneously detects, grades, and segments femoral fractures from x-ray images. The proposed method differs from other work in two aspects: (1) it provides a higher granularity on fracture type classification than prior art and (2) identifies the pixels of the image that led to that classification by providing the fracture segmentation mask.

The proposed model obtained a high AUC of 0.981 when detecting fractures. This AUC is comparable to results reported in prior work: 0.975 for Krogue et al [22] or 0.980 for Murphy et al [25], and to other methods implemented on our dataset, as shown in Table 3. When classifying the fracture according to location and severity, the method achieved an accuracy of 86.19%. Unfortunately, this metric cannot be compared directly to other work, as every study uses a different set of classes. For instance, Krogue et al [22] do not include subtrochanteric fractures, do not separate pertrochanteric into subclasses, and include the presence of arthroplasties or open reduction and internal fixation, which improve the accuracy of the system. The work of Murphy et al [25] does not distinguish between displaced and undisplaced femoral neck fractures or include pertrochanteric fractures. When evaluating the capacity of the model to segment fractures, the proposed model achieved a DSC of 0.77, demonstrating high concordance with human annotations.

A key question when developing artificial intelligence methods is the quality of the training data. We assessed it by implementing image classification models already published in the literature using our dataset. The AUCs obtained are consistent with prior studies. For instance, the DenseNet architecture, previously used by Krogue et al [22], achieved an AUC of 0.975 in their study and an AUC of 0.993 in our dataset. Similarly, Murphy et al [25] used an InceptionV3 architecture with AUCs in the range of 0.980, whereas our implementation achieved an AUC of 0.985. These results demonstrate that our dataset is comparable to that of prior work.

The number of annotated images is one of the key limitations of this work. Samples were selected randomly from clinical practice, thereby reflecting fracture prevalences. This resulted in a small number of undisplaced femoral neck and subtrochanteric fractures. Consequently, the model performs worse on these two types of fractures, as evidenced by the confusion matrix in Fig. 3. Future work will focus on semi-automated labeling methods to enrich the training set with fractures that appear with low probability on clinical fractures.

Although the images used in this study were obtained from a single institution, they were acquired using five different devices of two image modalities. This diversity in image acquisition enhances the likelihood that the model can generalize well to images from other devices. A formal study to evaluate models’ generalization capability is planned for future work.

Another limitation is the absence of evaluation of the method in a clinical setting. However, given its superior performance metrics compared to prior art and its unique ability to display the regions of the image that are fractured, it is expected that the proposed network will yield at least the same improvements in radiologists’ sensitivity and specificity as other methods when used as a second read.

A question remains regarding the comparative utility of segmentation versus the application of a bounding box or an arrow pointing to the fracture’s center. We hypothesize that segmenting the fracture line and associated bone fragments may offer greater confidence in automated diagnostics. Additionally, it may serve as a valuable educational tool for novice radiologists. Validation of these hypotheses will be explored in future studies.

To conclude, we have modeled femoral fracture detection and classification as an image segmentation problem using a single network. We have obtained better or comparable metrics to the state of the art, while including more fracture subtypes and having the unique capacity to display the region of the image where the fracture is present. Our method might enhance interpretability and confidence in the algorithm. Future work will include a formal evaluation by radiologists to confirm these anticipated benefits.

## Supplementary information

ELECTRONIC SUPPLEMENTARY MATERIAL

## Data Availability

The datasets used and analyzed during the current study are available from the corresponding author upon reasonable request.

## Abbreviations

AI: Artificial intelligence
AUC: Area under the curve
BIMCV: Medical Imaging Databank of the Valencia Region
CI: Confidence interval
CNN: Convolutional neural network
DSC: Dice similarity coefficient
DX: Digital radiography
IRB: Institutional Review Board
PACS: Picture Archiving and Communication System
ROC: Receiver operator characteristic
