# Supplementary material for: Classification and segmentation of hip fractures in x-rays: highlighting fracture regions for interpretable diagnosis
Source: Insights Imaging. 2025 Apr 15;16:86. doi: 10.1186/s13244-025-01958-y (PMC12000489; doi:10.1186/s13244-025-01958-y)
Supplement: Supplementary file 1 — ELECTRONIC SUPPLEMENTARY MATERIAL [file 13244_2025_1958_MOESM1_ESM.pdf]

# Classification and Segmentation of Hip Fractures in X-Rays: Highlighting Fracture Regions for Interpretable Diagnosis

## ELECTRONIC SUPPLEMENTARY MATERIAL

| | Model/Image size | 320 | 640 | 1024 | 1536 |
|---|---|---|---|---|---|
| AUC | *Nano* | 0.983 (0.006) | 0.984 (0.005) | 0.985 (0.008) | 0.986 (0.005) |
| | *Small* | 0.977 (0.007) | 0.987 (0.007) | **0.987 (0.006)** | 0.981 (0.004) |
| | *Medium* | 0.984 (0.004) | 0.985 (0.005) | 0.985 (0.007) | 0.978 (0.006) |
| Accuracy | *Nano* | 0.768 (0.019) | 0.778 (0.014) | 0.783 (0.016) | 0.774 (0.015) |
| | *Small* | 0.765 (0.023) | 0.784 (0.018) | **0.784 (0.017)** | 0.765 (0.020) |
| | *Medium* | 0.780 (0.017) | 0.781 (0.033) | 0.779 (0.011) | 0.768 (0.009) |
| DICE | *Nano* | 0.766 (0.020) | 0.778 (0.017) | 0.775 (0.013) | 0.773 (0.017) |
| | *Small* | 0.785 (0.010) | 0.779 (0.014) | 0.778 (0.010) | 0.768 (0.012) |
| | *Medium* | 0.775 (0.007) | 0.771 (0.023) | **0.781 (0.009)** | 0.779 (0.009) |

Supplementary Table S1: Yolo Validation
